# Retrospective Analysis of Spinal Radiographs for the Presence of Lumbosacral Transitional Vertebra in Patients with Axial Spondyloarthritis

**DOI:** 10.5152/ArchRheumatol.2025.11106

**Published:** 2025-06-23

**Authors:** Zerrin Kasap, Evren Er

**Affiliations:** Department of Physical Medicine and Rehabilitation, Giresun University Faculty of Medicine, Giresun, Türkiye

**Keywords:** Axial spondyloarthritis, low back pain, sacroiliitis, transitional vertebra

## Abstract

**Background/Aims:**

Axial spondyloarthritis (axSpA) is characterized by low back pain and sacroiliitis. It is important to exclude other causes of sacroiliitis before diagnosing axSpA. It was hypothesized that as one of the reasons for low back pain and sacroiliitis, the presence of lumbosacral transitional vertebra (LSTV) could lead to diagnostic confusion in axSpA. This study aimed to investigate the prevalence of LSTV in axSpA patients and whether LSTV caused any differences in disease characteristics compared to patients without LSTV.

**Materials and Methods:**

This was a retrospective study. Patients with axSpA who had available pelvic and lumbosacral spine radiographs and were over 18 years old were included. They were divided into 2 groups based on the presence of LSTV. These groups were compared in terms of age, sex, r-axSpA prevalence, biologic disease-modifying antirheumatic drugs (DMARDs) usage rates, and C-reactive protein (CRP)/erythrocyte sedimentation rate (ESR) levels. Likewise, patients with available disease-specific clinical scores (Ankylosing Spondylitis Disease Activity Score with C-reactive protein, Bath Ankylosing Spondylitis Disease Activity Index, Bath Ankylosing Spondylitis Functional Index, Bath Ankylosing Spondylitis Metrology Index [BASMI]) and those using biologic DMARDs were also divided into 2 groups based on the presence of LSTV and were analyzed accordingly.

**Results:**

A total of 130 patients (82 males, 48 females) were included. Ninety-five patients were using biologic DMARDs and 41 patients had available disease-specific clinical scores (only 19 had BASMI scores). The rate of presence of LSTV was 25.4% (n = 33). The most common type was Castellvi type 1b (39.4%). No significant differences were observed between axSpA patients with and without LSTV in terms of age, sex, r-axSpA prevalence, biologic DMARD usage, CRP/ESR levels, the number of different biologic DMARDs they had used, disease activity, physical function, and mobility.

**Conclusion:**

No diagnostic concerns were identified in axSpA patients with LSTV in this study. However, due to the high rate reported in this study, it is recommended that patients with LSTV undergo a more thorough evaluation prior to an axSpA diagnosis, with a diagnosis approach extending beyond simply meeting a set number of the Assessment in SpondyloArthritis international Society (ASAS) criteria.

**Main Points**

- No significant differences were observed between axSpA patients with and without LSTV in terms of age, sex, or any disease-related data.
- No diagnostic concerns were identified in axSpA patients with LSTV.
- Considering the high rate of LSTV reported in this study, patients with LSTV should undergo a more thorough evaluation prior to an axSpA diagnosis.

## Introduction

Spondyloarthritis (SpA) refers to a group of related rheumatic diseases consisting of ankylosing spondylitis (AS), psoriatic arthritis, inflammatory bowel disease-associated arthritis/spondylitis, and reactive arthritis.^1^ These are chronic inflammatory diseases that primarily affect the axial skeleton. The main clinical features of SpA include inflammatory low back pain, arthritis, enthesitis, uveitis, and dactylitis.^1^ While AS represents the diagnosis of radiographic axial SpA (r-axSpA) characterized by radiographic sacroiliitis, non-radiographic axial SpA (nr-axSpA) diagnosis is based on magnetic resonance imaging findings of the sacroiliac joint.^2^ However, diagnosing axSpA can be challenging since low back pain is one of the most common complaints in the general population, and no single disease feature has sufficient sensitivity and specificity to diagnose axSpA alone.^3,4^ The differential diagnosis includes a large number of diseases that can cause low back pain.^3^ Similar clinical and radiological characteristics are present in a variety of diseases.^5^ Before establishing a diagnosis of SpA in patients with low back pain and sacroiliitis, it is crucial to rule out other potential causes of sacroiliitis.

Lumbosacral transitional vertebra (LSTV) is a congenital spinal anomaly characterized by an enlarged transverse process of the last lumbar vertebra, which may be partially or fully fused with the first sacral segment.^6^ The prevalence has been reported as 3-10%.^7-10^ Lumbosacral transitional vertebra is recognized as a cause of mechanical low back pain.^9^ However, an association between LSTV and radiographic or magnetic resonance imaging (MRI)-detected sacroiliitis in patients with inflammatory low back pain has been demonstrated.^11^

Based on this information, it was hypothesized that the presence of LSTV could contribute to diagnostic uncertainly in axSpA. Moreover, if patients with LSTV were misdiagnosed with SpA, they would not respond to the SpA treatment. Although there are studies showing that LSTV is associated with radiographic/MRI sacroiliitis^11^ and sacroiliac dysfunction^12^ and that anatomically LSTV may cause structural changes in the sacroiliac joint,^13^ there is no study evaluating its relationship with the medication changes and potential misdiagnosis in patients with axSpA. Therefore, the aim was to investigate the prevalence of LSTV in patients classified as axSpA according to ASAS criteria, as well as whether this condition caused any differences in disease characteristics and response to treatment compared to patients without LSTV.

## Materials and Methods

This study was conducted in accordance with the Declaration of Helsinki and approved by the ethical committee of the Giresun Education and Research Hospital (approval no. 18.02.2023/05). Due to the retrospective nature of the study, informed consent was not obtained.

This study was a retrospective descriptive study. Patients who applied to the hospital between July 1, 2022 and July 1, 2023 were retrospectively scanned with M02 postinfective and reactive arthropathies, M07 psoriatic and enteropathic arthropathies, M45 ankylosing spondylitis, and M46 other inflammatory spondylopathies ICD codes. All patients aged 18 years and over with axSpA and whose pelvic and lumbosacral spine radiographs were available were included in the study. Histories of other systematic diseases, additional rheumatologic disease, trauma, and lumbar or lower extremity surgery were exclusion criteria.

The sociodemographic and clinical features were recorded, including age, sex, presence of LSTV and its type if present, presence of r-axSpA, medications, the latest CRP (C-reactive protein) and ESR (erythrocyte sedimentation rate) levels for all patients, and disease-specific clinical scores (ASDAS-CRP, BASDAI, BASFI, BASMI) if available.

Based on the presence of LSTV, the patients were divided into 2 groups: those with and without LSTV. These groups were compared in terms of age, sex, r-axSpA prevalence, biologic DMARD usage rates, CRP, and ESR levels. Likewise, patients using biologic DMARDs and those with available disease-specific clinical scores (ASDAS-CRP, BASDAI, BASFI, BASMI) were also divided into 2 groups based on the presence of LSTV and analyzed accordingly. Additionally, analyses of disease activity parameters of the patients using biologic DMARD were performed regarding the presence of LSTV.

The pelvic and lumbosacral spine radiographs were evaluated by 2 different physicians. In case of any discrepancies between results, they were re-evaluated by both physicians together. Pelvic radiographs were assessed in accordance with the Modified New York criteria for the diagnosis of r-axSpA. Presence of bilateral stage 2 or unilateral stage 3-4 sacroiliitis were evaluated r-axSpA.^14^

In this study, LSTV was evaluated in lumbosacral radiographs using the Castellvi classification. In this classification, LSTV is defined in 4 different types based on the enlargement of the transverse process of the final lumbar vertebra, as well as the presence of pseudoarticulation or fusion. The first 3 types are further subdivided into subtypes “a” and “b” regarding being unilateral or bilateral. While type 1a refers to unilateral enlargement of the transverse process only (≥19 mm), type 3b refers to bilateral fusion. Additionally, type 4 refers to unilateral type 2 and contralateral type 3.^15-17^ Also, for sacroiliitis grading and classification of r-axSpA, the New York criteria were used.^14^

For detection of disease activity scores, the ASDAS-CRP score, which evaluates back pain, morning stiffness, patient global, peripheral pain/swelling, and CRP levels,^18^ and the BASDAIscore, which evaluates fatigue/tiredness, neck/back/hip pain, peripheral joint pain and swelling, localized tenderness, and morning stiffness, were used.^19^ These scores provide a comprehensive assessment of the patient’s condition, allowing for more tailored treatment plans and better monitoring of disease progression. Furthermore, the BASFI score was used to evaluate the patient’s functionality in daily living activities,^20^ and the BASMI score, which evaluates the patient’s mobility by assessing metrology of lateral lumbar flexion, tragus-to-wall distance, lumbar flexion, maximal intermalleolar distance, and cervical rotation. In this study, BASMI_2 _method was used.^21,22^

### Statistical Analysis

Categorical variables are expressed as percentages and numbers, and continuous variables are expressed as median with interquartile range (IQR) or mean ± SD. The Kolmogorov-Smirnov test was utilized to assess whether the numerical data were normally distributed. The Mann-Whitney *U* test was used to compare any 2 groups with non-normally distributed data, or when the sample size was less than 10. The independent sample t test was used to compare any 2 groups that had a normal distribution. In addition, Pearson’s chi-squared test was used to compare categorical variables. All statistical analyses were performed using IBM SPSS version 23.0 (IBM SPSS Corp.; Armonk, NY, USA). A *P*-value of less than .05 was considered statistically significant.

## Results

**Figure 1.**
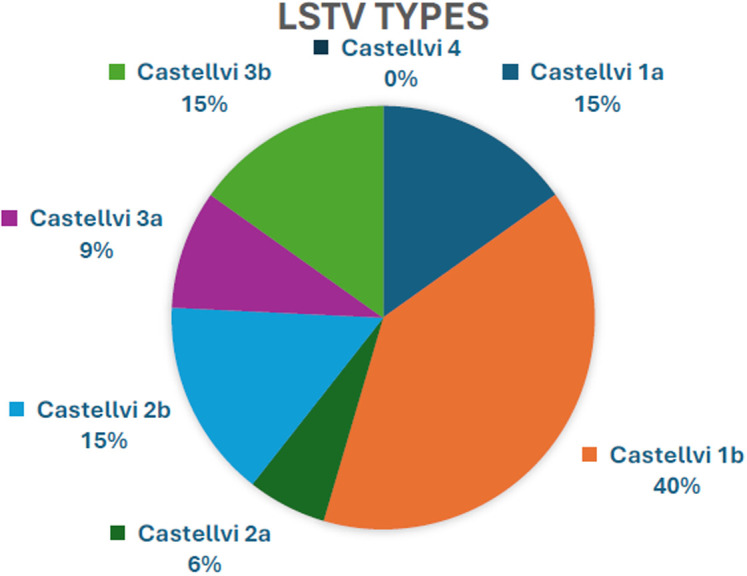
Percentages of lumbosacral transitional vertebra types.

A total of 130 patients (82 males, 48 females) who were classified as axSpA in accordance with ASAS criteria were included. The mean age was 44.84 ± 12.08 years. About 66.2% of the patients (n = 86) had r-axSpA. The rate of usage of biologic DMARDs was 73.1% (n = 95). The rate of presence of LSTV was 25.4% (n = 33). Among patients with LSTV, 69.7% (n = 23) were male. The distribution of LSTV types is shown in Figure 1, with Castellvi type 1b being the most common (39.4%).

The comparisons between the patients with and without LSTV are shown in Table 1. No statistically significant difference was observed regarding age, sex, r-axSpA rate, biologic DMARD usage rate, CRP levels, or ESR levels.

Among patients using biological DMARDs (n = 95), there was no significant difference in the number of different agents used between those with and without LSTV (Table 2). This finding was consistent in the subgroup of patients with r-axSpA using biological DMARDs (n = 67), where the number of different agents used was also similar between the 2 groups (Table 3).

Due to the retrospective design of the study, disease-specific clinical scores were not available for all patients. ASDAS-CRP, BASDAI, and BASFI scores were available for 41 patients, while BASMI scores were available for only 19 patients. When these groups were also divided into 2 groups regarding the presence of LSTV, no statistically significant differences were found in terms of disease activity, functional status, or mobility (Table 4).

There were 37 patients using biologic DMARDs with available disease-specific clinical scores. In order to find out the differences in treatment response, the analyses of disease activity parameters of the patients using biologic DMARD were performed regarding the presence of LSTV. There were no statistically significant differences in treatment response (Table 5).

## Discussion

The main hypothesis of this study was that the axSpA and r-axSpA might be potential misdiagnoses since LSTV is also associated with MRI and radiographic sacroiliitis. For this purpose, the present study evaluated the presence of lumbosacral transitional vertebrae in patients with axSpA and examined their relationship with disease-related data.

A total of 130 patients with axSpA who met ASAS criteria were included. The LSTV rate was found to be 25.4%. The LSTV rate in the study was higher than that in other prevalence studies.^7-10^ No difference was found in terms of age, sex, r-axSpA prevalence, biologic DMARD usage, CRP/ESR levels, the number of different biologic DMARDs they had used, disease activity, physical function, and mobility.

The most common LSTV types differ in studies examining LSTV types in the general population [type 1,^23^ type 2^24^ or type 3^25^]. Contrary to the literature indicating that the most common types of LSTV associated with low back pain are type 2-4, in this study, the most common type was type IB, similar to the study conducted by Carvajal et al.^11^ Also, the assessment of the gender distribution among patients with LSTV revealed a higher prevalence of male patients (69.7%), which contrasts with the findings of Dzupa et al.^26^

When ASAS criteria are considered, the presence of sacroiliitis and good response to non-steroidal anti-inflammatory drugs (NSAIDs) in patients younger than 45 years of age with low back pain lasting longer than 3 months is significant for SpA.^27^ Additionally, clinical and radiological criteria of Modified New York Criteria for AS consist of inflammatory back pain lasting longer than 3 months, limitation of lumbar joint range of motion and chest expansion, and radiological demonstration of sacroiliitis.^14^ In a study conducted by Carvajal et al,^11^ a relationship was found between LSTV and sacroiliitis.^11^ Accordingly, in LSTV patients, pain that is intense in the morning and relieved by NSAIDs and the presence of sacroiliitis together with it may be misleading for the diagnosis of axSpA when looking at the ASAS and Modified New York criteria. For this reason, there are various criticisms in previous studies regarding the modification of ASAS and modified New York criteria.^28,29^ In the Caspar criteria, since there are objective criteria such as the presence of psoriasis/family history, nail dystrophy, dactylitis, and bone proliferation in the bones adjacent to the joint, it is not expected to cause confusion in patients with LSTV.^30^

In the initial phase of axSpA treatment, NSAIDs are administered. For patients unresponsive to these, biologic agents, including tumor necrosis factor inhibitors, janus kinase inhibitors, and interleukin-17 inhibitors, are recommended.^31^ An important tool in the management of SpA treatment is the ASDAS (Ankylosing Spondylitis Disease Activity Score), which places significant emphasis on back pain intensity as a core component, alongside the Bath Ankylosing Spondylitis Disease Activity Index (BASDAI).^27,32^ While the scores are primarily intended to reflect inflammatory pain, back pain is a subjective symptom; thus, other causes of back pain in patients may lead to inaccuracies in calculations. Non-steroidal anti-inflammatory drugs are also used in the treatment of LSTV patients.^33^ However, since the condition will persist as a mechanical disorder, it is not expected that the symptoms will completely improve with these drugs. In this study, there were patients using biologic DMARDs in the group with LSTV, and the proportion of patients using biologic DMARDs between the 2 groups (with/without LSTV) was statistically similar. This similarity suggests that treatment in patients with LSTV provides similar benefits as in the other group and does not raise suspicion of misdiagnosis of axSpA. Additionally, if LTSV led to a misdiagnosis in the included patients, it would be expected that patients with LSTV would use a greater number of different biologic DMARDs due to unresponsiveness to biologic treatment, but in this study, no difference was found between the 2 groups in terms of the number of patients using biologic DMARDs and in terms of drug changes in patients using biologic DMARDs. When only patients with sacroiliitis were evaluated, no difference was found between the 2 groups in terms of drug changes in patients using biologic DMARDs.

In spondyloarthropathies, in addition to sacroiliitis, Romanus and Anderson lesions and syndesmophytes can be observed radiologically.^34,35^ Sacroiliitis and lumbar fusion can be observed in LSTV patients, but other imaging findings observed in SpA patients are not expected.^11^ Radiological findings may be insufficient in the early disease period and may cause diagnostic confusion.^36^ Since the study was designed retrospectively, additional lumbar MRI examinations could not be performed on the patients. In this study, the groups (with/without LSTV) were similar in terms of percentage of r-axSpA.

Since it was assumed that the axSpA and r-axSpA might be potential misdiagnosis since LSTV is also associated with MRI and radiographic sacroiliitis,^11^ it was hypothesized that patients with LSTV in each group (all axSpA patients using biologic DMARD and the r-axSpA patients using biologic DMARD) would need more DMARD switching than patients without LSTV. In order to explore this, in each of these groups the number of how many different biologic DMARDs they had used were analyzed regarding the presence of LSTV. Nonetheless, there was no statistically significant difference to support this hypothesis. Biologic DMARDs used in the treatment of SpA effect by suppressing inflammation and are not expected to alleviate mechanical pain associated with LSTV. Since, in case of misdiagnosis, patients are unlikely to respond to treatment,^31^ re-evaluation of the diagnosis and comorbidities is suggested in ACR/EULAR recommendations if the treatment is unsuccessful.^27^ Therefore, it is expected that SpA patients with misdiagnosis would switch to more biologic DMARDs due to the treatment’s unresponsiveness. However, in this study, there was no statistically significant difference between the 2 groups in terms of biologic DMARD switching. This result does not support the hypothesis and suggests that there is no diagnostic confusion.

Questionnaires (ASDAS-CRP, BASMI, BASFI, and BASMI) are commonly used indices in the follow-up of patients with spondyloarthropathy. With appropriate treatment, a decrease in these index scores is expected.^32^ If no decrease is observed, it may be necessary to re-evaluate the diagnosis and treatment. In this study, when the SpA patients were divided into groups based on with and without LSTV, no statistically significant difference was found in any of the questionnaire scores. Based on these findings, it was thought that the clinical status of both groups is similar and that they benefit or do not benefit from the treatment at a similar rate. In other words, the presence of LSTV does not raise any diagnostic confusion. However, since the index scores were obtained retrospectively and represent only a specific period of the disease, they may not fully reflect the treatment responses. Therefore, interpreting the results solely based on these scores may not be entirely objective.

The primary limitation of this study was its retrospective design. Because of its design, important disease-related data such as age at diagnosis, disease duration, clinical features of SpA such as peripheral arthritis, enthesitis, and extra-articular manifestations and disease activity scores could only be partially available for some of the patients; the patients who had involuntarily incorrect ICD codes could not be involved; and the treatment comparison between patient groups with and without LSTV who received biological treatment was restricted to only the number of different biological agents used. Additionally, another limitation was that since the main focus of the study was the effect of LSTV on radiographic changes, the effect of LSTV on MRI sacroiliitis findings was not evaluated and did not incorporate MRI imaging. Lastly, there was no potential bias in the study regarding the sample selection, since all the patients who met the inclusion criteria in the stated time period were included. However, regarding these data being collected from only 1 center, it may not be possible to generalize to all axSpA patients.

In conclusion, although no diagnostic concerns were identified in axSpA patients with LSTV in this study, it is recommended that patients with LSTV undergo a more thorough evaluation prior to an axSpA diagnosis, with a diagnosis approach extending beyond simply meeting a set number of ASAS criteria. In future studies, it will be more valuable to evaluate disease activity scores, treatment responses, and other findings of spondyloarthritis (enthesitis, uveitis, arthritis, etc.), including subtypes of LSTV, in AS patients with LSTV.

## Figures and Tables

**Table 1.** Comparisons of Demographic Characteristics and Disease-related Data

		**Patients with LSTV** **n = 33 (100%)**	**Patients without LSTV** **n = 97 (100%)**	***P***
**Age (mean ± SD)**		45.48 ± 10.30	44.62 ± 12.67	.723^t^
Sex	Female n = 48	10 (30.3%)	38 (39.2%)	.362^x2^
	Male n = 82	23 (69.7%)	59 (60.8%)	
r-axSpA	+ n = 86	21 (63.6%)	65 (67.0%)	.723 ^x2^
	− n = 44	12 (36.4%)	32 (33.0%)	
Biologic DMARDs usage	+ n = 95	24 (72.7%)	71 (73.2%)	.958 ^x2^
	− n = 35	9 (%27.3)	26 (26.8%)	
CRP [median (IQR)]		4.02 (8.35)	2.84 (7.35)	.187 ^m^
ESR [median (IQR)]		17 (16.5)	18 (21)	.387 ^m^

CRP, C-reactive protein; DMARDs, disease-modifying antirheumatic drugs; ESR, erythrocyte sedimentation rate; IQR, interquartile range; LSTV, lumbosacral transitional vertebra; m, Mann-Whitney *U* test; r-ax-SpA, radiographic axial spondyloarthritis; t, Student *t* test; x^2^, Pearson’s chi-square.

**Table 2.** Comparisons of the Number of Different Biologic Disease-Modifying Antirheumatic Drugs

	**Patients with LSTV** **(n = 24)**	**Patients Without LSTV** **(n = 71)**	***P***
Number of different biologic DMARDs [median (IQR)]	1.00 (1.00)	2.00 (1.00)	.280^m^

DMARDs, disease-modifying antirheumatic drugs; IQR, interquartile range; LSTV, lumbosacral transitional vertebra; m, Mann-Whitney *U* test.

**Table 3.** Comparisons of the Number of Different Biologic Disease-Modifying Antirheumatic Drugs in Radiographic Axial Spondyloarthritis Patients

	**Patients with LSTV** **(n = 18)**	**Patients without LSTV** **(n = 49)**	***P***
Number of different biologic DMARDs [median (IQR)]	1.00 (1.00)	1.00 (1.00)	0.326^m^

DMARDs, disease-modifying antirheumatic drugs; IQR, interquartile range; LSTV, lumbosacral transitional vertebra; m, Mann-Whitney *U* test.

**Table 4.** Comparisons of Disease-Related Scores

	**Patients with LSTV** **n = 11**	**Patients without LSTV** **n = 30**	***P***
ASDAS-CRP (mean ± SD)	2.33 ± 1.16	2.38 ± 1.39	.911 ^t^
BASDAI (mean ± SD)	3,39 ± 2.61	3.58 ± 2.66	.838 ^t^
BASFI [median (IQR)]	2.40 (4.60)	2.80 (5.23)	.627 ^m^
	**Patients with LSTV** **n = 5**	**Patients Without LSTV** **n = 14**	***P***
BASMI [median (IQR)]	1.70 (2.90)	2.45 (2.1)	.517 ^m^

ASDAS-CRP, Ankylosing Spondylitis Disease Activity Score with C-reactive protein; BASDAI, Bath Ankylosing Spondylitis Disease Activity Index; BASFI, Bath Ankylosing Spondylitis Functional Index; BASMI, Bath Ankylosing Spondylitis Metrology Index; IQR, interquartile range; LSTV, lumbosacral transitional vertebra; m, Mann-Whitney *U* test; t, Student t test.

**Table 5.** Comparisons of Disease Activity Parameters of the Patients Using Biologic Disease-Modifying Antirheumatic Drugs Regarding the Presence of Lumbosacral Transitional Vertebra

	**Patients with LSTV n = 11**	**Patients Without LSTV n = 26**	***P***
CRP levels [median (IQR)]	3.50 (4.89)	3.73 (11.55)	.756
ESR levels [median (IQR)]	17.00 (16.00)	20.50 (21.00)	.635
ASDAS-CRP scores (mean ± SD)	2.32 ± 1.16	2.53 ± 1.42	.666
BASDAI scores (mean ± SD)	3.39 ± 2.61	3.88 ± 2.66	.608
BASFI scores [median (IQR)]	2.40 (4.60)	2.85 (5.48)	.441

ASDAS-CRP, Ankylosing Spondylitis Disease Activity Score with CRP; BASDAI, Bath Ankylosing Spondylitis Disease Activity Index; BASFI, Bath Ankylosing Spondylitis Functional Index; CRP, C-reactive protein; ESR, erythrocyte sedimentation rate; IQR, interquartile range; LSTV, lumbosacral transitional vertebra.

## Data Availability

The data that support the findings of this study are available on request from the corresponding author.
